# Dopamine disruption increases negotiation for cooperative interactions in a fish

**DOI:** 10.1038/srep20817

**Published:** 2016-02-08

**Authors:** João P. M. Messias, José R. Paula, Alexandra S. Grutter, Redouan Bshary, Marta C. Soares

**Affiliations:** 1 CIBIO, Centro de Investigação em Biodiversidade e Recursos Genéticos, Universidade do Porto, Vairão, Portugal; 2 MARE - Centro de Ciências do Mar e do Ambiente, Laboratório Marítimo da Guia, Faculdade de Ciências da Universidade de Lisboa, Portugal; 3 School of Biological Sciences, The University of Queensland, Brisbane, Queensland, Australia; 4 Université de Neuchâtel, Institut de Biologie, Eco-Ethologie, Neuchâtel, Switzerland

## Abstract

Humans and other animals use previous experiences to make behavioural decisions, balancing the probabilities of receiving rewards or punishments with alternative actions. The dopaminergic system plays a key role in this assessment: for instance, a decrease in dopamine transmission, which is signalled by the failure of an expected reward, may elicit a distinct behavioural response. Here, we tested the effect of exogenously administered dopaminergic compounds on a cooperative vertebrate’s decision-making process, in a natural setting. We show, in the Indo-Pacific bluestreak cleaner wrasse *Labroides dimidiatus,* that blocking dopamine receptors in the wild induces cleaners to initiate more interactions with and to provide greater amounts of physical contact to their client fish partners. This costly form of tactile stimulation using their fins is typically used to prolong interactions and to reconcile with clients after cheating. Interestingly, client jolt rate, a correlate of cheating by cleaners, remained unaffected. Thus, in low effective dopaminergic transmission conditions cleaners may renegotiate the occurrence and duration of the interaction with a costly offer. Our results provide first evidence for a prominent role of the dopaminergic system in decision-making in the context of cooperation in fish.

Humans excel in their ability to cooperate among unrelated individuals^1^ but it is clear that there is also enormous variation between other species with respect to their cooperative tendencies. A recent major research focus is to understand the mechanistic basis of cooperative behaviour, particularly the cognitive and physiological processes underlying decision-making^2^,^3^,^4^. Vertebrate brain structures involved in social decision-making are highly conserved. Most importantly, all vertebrates have a so-called social decision-making network, which consists of the social behaviour network and the mesolimbic reward system^5^,^6^. This network appears to be highly sensitive to the dopaminergic system^5^,^7^, making dopamine a prime candidate for the modulation of cooperative behaviour.

Dopamine (DA) is a neurotransmitter involved in a variety of neurochemical and neurohormonal actions that affect and modulate animal behaviour and cognition^4^,^6^. Dopamine is involved in reward and risk assessment, behaviour reinforcement^8^,^9^, and anticipatory responses to reward-associated stimuli^8^ as its release signals the outcome of an action as appetitive or aversive^10^,^11^. Thus, DA is key to associative learning^12^. First, DA signals the delivery of an unexpected outcome (reward or punishment) which is usually preceded or paired with specific stimuli^13^. Later, through repeated encounters, individuals learn to associate the outcome with the preceding stimuli, and the dopaminergic response progressively shifts to this earlier event-predicting stimuli rather than responding to the outcome itself^11^,^14^,^15^,^16^. This gradually enables animals to anticipate outcomes in current interactions by recalling previously learned associations, which results in appropriate decision-making^17^. Moreover, DA signalling suffers a depression (DA transmission decreases momentarily) whenever the event happens contrarily to the prediction and the expected outcome fails to occur^18^. This decrease may elicit a distinct behavioural response: for example, in humans, the omission of an expected reward can lead to emotional distress^19^, while in other mammals, birds and teleost fish it may induce aggressive behaviour^20^,^21^,^22^. Nevertheless, signalling environmental changes is key for learning and decision-making, as these allow for an evaluation of the behavioural adjustments needed in order to achieve the expected outcome once again^23^. As such, anticipation is crucial for deciding between different courses of action available^18^, as different options entail uncertain final outcomes. A prime context in which correct anticipation is crucial is cooperation between unrelated individuals that is based on investments. The classic theoretic game model to describe such cooperation is the iterated prisoner’s dilemma^24^. In this 2-player game, mutual cooperation yields higher payoffs than mutual defection but defecting yields a higher payoff than cooperating, independently of the partner’s action. Thus, there are incentives both to cooperate and to defect, and an individual’s best decision will depend on the partner’s previous strategy^25^. Similar conflicting incentives exist in many other potentially cooperative interactions^26^. A good example is the marine cleaning mutualism involving the Indo-Pacific bluestreak cleaner wrasse *Labroides dimidiatus*. As summarised elsewhere^27^, these territorial cleaner fish remove ectoparasites from visiting ‘client’ reef fish. Interactions are best described as a repeated game; clients are estimated to visit cleaning stations typically 5–30 times per day, with maximal estimates above 100 visits^28^. A conflict of interest exists because cleaners prefer to eat client mucus, which constitutes cheating as it is detrimental to the client. Cheating is visible to the human observer through clients performing body jolts in response to cleaner wrasse mouth contacts^27^. As a consequence of cleaner wrasse food preferences, clients have to make cleaners feed against their preference to obtain a good service. How this is achieved depends on the clients’ strategic options in this repeated game. For predatory clients, the mere threat of reciprocation (trying to eat a cheating cleaner) is apparently enough to cause high service quality, while non-predatory client species either punish cleaners through aggressive chasing or leave and switch to a different cleaner for their next inspection, which constitutes the threat of departure^27^,^29^. In response, cleaners flexibly adjust their cheating frequency to a variety of parameters, which include client’s control mechanisms, the presence of bystanders, the presence of a co-inspecting cleaner partner, the client’s value as a food source and also the cleaner’s own physiological state^27^,^30^,^31^,^32^. Furthermore, cleaners can improve their service quality by providing a form of physical contact (known as tactile stimulation or massages) to clients, touching them with their pectoral and (especially) pelvic fins. Cleaners use tactile stimulation in a variety of contexts but usually when the outcome of the interaction is not certain: to build relationships with new clients, to reconcile after a cheating event, to prolong interactions with clients about to leave and as a pre-conflict management strategy with predators^33^,^34^. Clients apparently benefit from receiving tactile stimulation as it lowers baseline and acute stress levels (i.e. cortisol levels^35^). Thus, in marine cleaning mutualisms, two elements of behavioural negotiation are used by partners to resolve the conflict over cooperative payoffs: a) the use of threats (reciprocity or departure) and b) the use of tactile stimulation to encourage clients to stay at cleaning stations as a type of negotiation^29^. Overall, game theory has successfully been used to predict and explain partner control mechanisms in this system^36^. Regarding cleaner wrasses’ behavioural adjustments, game models should consider how physiological constraints (for example, the existence of stressed cleaners^32^) may limit the expression of some of these decision rules.

Here, we aimed to investigate the relevance of the dopaminergic system for the cleaners’ service quality during cleaning interactions, and how these individuals respond to changes of perception elicited by DA level shifts. Only a few studies have examined the role of the DA system on the modulation of fish behaviour, mostly on locomotor activity^37^, brain responses to light and hydrostatic pressure^38^, feeding behaviour^39^, coping with unpredictability^40^, learning and nicotine^41^, gene expression and neuroendocrine signalling^42^,^43^,^44^,^45^, and learning performance in a cooperative context^46^. Only some of the above-cited studies employed drugs aimed at the Dopamine D1 and D2 receptors, that were previously developed for mammals, which were successfully used in fish to test for putative effects on behaviour or gene expression^37^,^46^. For example, in cichlids, the effects caused by the use of a non-selective DA agonist that activates both D1 and D2 receptors on locomotor activity were blocked by the D1 antagonist (SCH-23390) but not by the D2 antagonist (metoclopramide). Also, the effects of several D1 and D2 related drugs produced distinct neuroendocrine and brain expression responses^42^,^43^,^44^,^45^. Using cleaners, Messias and colleagues^46^ showed that there is a direct involvement of the D1 receptor pathways on their natural ability to learn. As in these previous studies, we exogenously administered a D1 receptor agonist (D1a - SKF38393), an antagonist (D1an - SCH23390), a D2 receptor agonist (D2a - Quinpirole) and an antagonist (D2an - Metoclopramide), as well as a control (saline) to female cleaner wrasses *in situ*. As this mutualistic system occurs in a biological market^27^,^47^, efficient dopaminergic transmission could play a role in the modulation of cleaners’ willingness to negotiate with clients over the occurrence and duration of interactions as well as cleaners’ willingness to cooperate rather than cheating^27^. High increases in DA transmission via administration of agonists are connected with pathological gambling^48^ and excessive risk-taking^49^. Hence, we predict D1a and D2a to decrease cleaners’ cooperative investment levels and increase their cheating frequencies. Since D2 receptors can also be found pre-synaptically (i.e. as auto-receptors) in some areas of the brain, it is also possible that D2 stimulation leads to risk-avoidance behaviour by overstimulating the pre-synaptic receptors^50^. Similarly, DA receptor blockade induces risk-avoidance behaviour through an increase in sensitivity to negative stimuli^49^,^51^,^52^. We thus also predict that DA antagonists would cause cleaners to seek clients to clean more and provide more tactile stimulation to entice clients to stay longer, with the possibility that blocking the D2 autoreceptors might lead to abnormal DA transmission. Regarding cheating by cleaners, a perceived reduction in the probability of expected outcomes would mean a reduced ability to maintain the interaction with clients and a lower likelihood to obtain food. Such perception may, either lead to an extension of negotiation, where high rates of tactile stimulation lead to a reduction of cheating, or to just abandoning negotiation – with cleaners foraging as much and as quickly possible, which would mean immediate cheating by feeding on clients’ mucus^53^.

## Results

The five compounds administered consisted of a D1 receptor agonist SKF38393 (D1a) and antagonist SCH23390 (D1an), a D2 receptor agonist Quinpirole (D2a) and antagonist Metoclopramide (D2an), and a saline solution as a control. Using *Statistica* 12 software, Kruskal-Wallis ANOVA tests were performed to detect differences between treatments in each behavioural variable, and Dunn’s Post-Hoc tests were applied to compare each treatment against the control group which include a Bonferroni adjustment to account for multiple comparisons^54^.

### Dopamine effects on the likelihood to engage in cleaning behaviour

**Figure 1:**
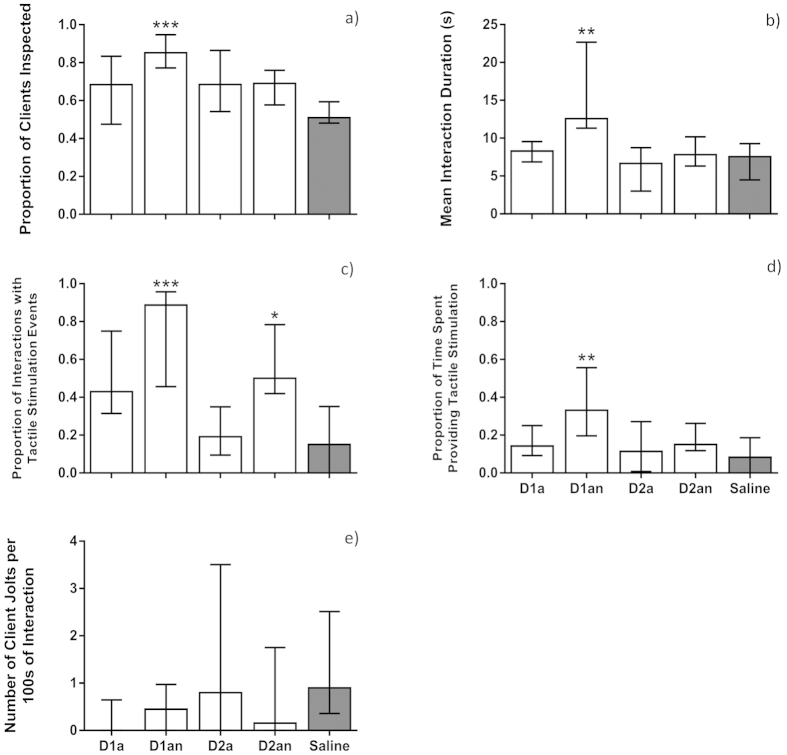
The effect of D1 agonist (D1a) and antagonist (D1an), D2 agonist (D2a) and antagonist (D2an) on cleaners’ likelihood to engage in cleaning behaviour measured by (**a**) the proportion of clients inspected (calculated as the total number of clients inspected/total number of visits) and (**b**) the mean interaction duration (total time of interaction/total number of interactions); cleaners’ cooperative investment measured by: (**c**) the proportion of interactions with tactile stimulation events (frequency of clients inspected where tactile stimulation occurred/total number of interactions), (**d**) the proportion of time cleaners spent providing tactile stimulation (total tactile stimulation duration/total interaction duration); and (**e**) cleaner wrasse cheating levels measured by the frequency of client jolts per 100 seconds of inspection. Medians and interquartile ranges are shown. Significant values are shown above bars: *<0.05; **<0.01; ***<0.001 and refer to Dunn’s Post-Hoc tests affecting each dopamine treatment against the reference (saline) group, for a total sample size of 10 individuals.

Cleaner wrasses treated with D1an inspected a significantly higher proportion of clients when compared with the control group (Kruskal-Wallis ANOVA, H (4, N = 50) = 17.4435, *p* = 0.0016; Dunn’s Post-Hoc test: D1an vs Saline, *p* = 0.0001, Fig. 1A), whereas other treatments did not differ from the control group (Dunn’s Post-Hoc test: D1a vs Saline, *p* = 0.4000; D2a vs Saline, *p* = 0.1451; D2an vs Saline, *p* = 0.2764, Fig. 1A). Cleaner wrasses treated with D1an also had on average longer interactions with clients compared with the control group (Kruskal-Wallis ANOVA, H (4, N = 50) = 18.2820, *p* = 0.0011; Dunn’s Post-Hoc test: D1an vs Saline, *p* = 0.0025, Fig. 1A), whereas other treatments did not differ from the control group (Dunn’s Post-Hoc test: D1a vs Saline, *p* = 0.4000; D2a vs Saline, *p* = 0.4000; D2an vs Saline, *p* = 0.4000, Fig. 1B).

### Dopamine effects on tactile stimulation of clients

Cleaner wrasses treated with either D1an or D2an had a higher proportion of interactions in which they provided tactile stimulation to their clients compared to the control group (Kruskal-Wallis ANOVA, H (4, N = 50) = 23.47111, *p* = 0.0001; Dunn’s Post-Hoc test: D1an vs Saline, *p* = 0.0004; D2an vs Saline, *p* = 0.0111, Fig. 1C) whereas other treatments did not differ from the control group (Dunn’s Post-Hoc test: D1a vs Saline, *p* = 0.0856; D2a vs Saline, *p* = 0.4000, Fig. 1C). Cleaner wrasse treated with D1an spent a greater proportion of inspection time providing tactile stimulation to their clients (Kruskal-Wallis ANOVA, H (4, N = 50) = 11.1371, *p* = 0.0251; Dunn’s Post-Hoc test: D1an vs Saline, *p* = 0.0082, Fig. 1D), whereas other treatments did not differ from the control group (Dunn’s Post-Hoc test: D1a vs Saline, *p* = 0.4000; D2a vs Saline, *p* = 0.4000; D2an vs Saline, *p* = 0.4000, Fig. 1D).

### Dopamine effects on cleaner wrasses cheating levels

Client jolt frequency in all dopamine treatments did not differ from the control group (Kruskal-Wallis ANOVA, H (4, N = 50) = 4.76234, *p* = 0.3126, Fig. 1E).

## Discussion

We provide experimental evidence that the dopaminergic system is linked to the decisions of cleaner wrasses during interactions with client reef fish. Decreasing DA transmission was effective, particularly with respect to the D1 type receptor, while increasing effective dopamine transmission did not yield measurable effects. This latter result agrees with previous studies suggesting that increases in dopaminergic transmission are not as relevant to decision making based on previously made associations as it is during the learning process^13^. Decreases in DA transmission signal an outcome that is worse than predicted^11^, which in the case of cleaners would mean a reduced likelihood to obtain food during a cleaning interaction or higher chances of being punished (by being chased or the client leaving). In game-theoretical terms, physiological evidence suggests that dopamine affects an individual’s perception of the payoffs associated with each potential action. The key question was then how cleaners would respond to changes in payoff perception in a repeated game with respect to their effort to make the interaction happen or to prolong it and how this would affect their level of cooperative foraging. Our results show that blocking DA receptors made cleaners seek more interactions and increase tactile stimulation to clients (both duration and frequency), i.e. the behaviours that cleaners can use to negotiate the occurrence and duration of interactions with clients^27^. In contrast, levels of cooperative foraging and hence cheating rates remained unaffected by our experimental manipulations. In other words, reductions in normal DA tone lead cleaners to renegotiate the occurrence and duration of the interaction using a ‘costly offer’ instead of reducing overall cheating rates. The lack of change in cheating rates was surprising, given that theoretical considerations would predict either an increase or a decrease^51^,^53^. A new model that captures the specifics of cleaning interactions may help to establish whether our measured effects of dopamine on cleaners would be optimal under natural conditions. Such a model could also investigate how far changes in the perception of repetitiveness probability would produce similar effects or not. Moreover, future research should test for the role of dopaminergic pathways on cleaner wrasse decision-making under particular conditions in which the temptation to cheat is higher, for example in laboratory “eating against preference” contextual tasks (alone or in pairs).

The blockade of dopamine receptors (both D1 and D2) appeared to cause cleaners to behave as if clients were permanently in dispute about the value of being serviced (increasing sensitivity to negative stimuli). In this case, the use of tactile stimulation agrees with previous findings, which revealed that cleaners provide tactile stimulation to manipulate client decisions, to appease predators, to build up relationships and to reconcile after a conflict^33^,^34^. Tactile stimulation is a costly form of negotiation as it is incompatible with foraging. Thus, changes in the dopaminergic system appear to be responsible for the modulation of cleaner wrasses’ perception (i.e. anticipation) concerning the expected reward and the predicted costs/risks involved in any given decision when dealing with clients (i.e. the client is viewed as more likely to leave). To the best of our knowledge, the present study represents the first examination of the effect of exogenous dopaminergic compounds’ administration in a cooperative decision-making process tested in a natural setting. The results fit laboratory studies on the role of dopamine in the modulation of risky based decision-making in humans^55^,^56^ and rats^51^. For instance, individuals with lower levels of dopamine in the prefrontal cortex tend to risk less in a gambling task^57^. Another example of effects of depletion of dopaminergic transmission involves untreated patients with Parkinson’s disease which are more sensitive to negative or uncertain outcomes (risk aversion)^55^,^56^.

Blockade of dopamine D2 receptors caused an increase of tactile stimulation events but not the amount of time spent providing it, while D1 blockade produced a stronger impairment on cleaner wrasses’ overall behaviour (which also included the frequency and time spent providing physical contact to clientele). Indeed, there are general differences in the affinity of DA to each of these receptors (as shown for mammals): for instance, D2-like receptors have a 10-to-100 fold greater affinity than the D_1_-like family, with the D1a being reported to have the lowest affinity for DA^58^,^59^. Moreover, because the D1-like receptors are solely found postsynaptically, while the D2-like receptors are the predominant type of autoreceptor being found both pre- and postsynaptically^58^, it is expected that the pathways involved in the function of both receptor types are likely distinct. D2 receptors are able to induce a negative feedback regulation that may inhibit DA neuron firing, synthesis and release^60^, functioning as a control mechanism, and D1 receptors, in contrast, play a direct stimulatory role. Also, some studies have referred to opposing but sometimes complementary roles played functionally by D1 and D2 receptors. This is in agreement with our results which suggest that both receptors regulate these behavioural trade-offs, yet with different magnitudes, but mostly that these receptors manage the perception of feedback received by the focal animal during interactions, with D1 receptors having the main modulatory role^60^. Another potential explanation would arise from a putative unexpected effect of the D2 blockade on D1 receptors, which is less likely to occur due to differences in affinity between both receptor types and the selectivity of the compounds used^37^,^46^.

The absence of an effect when D1 and D2 selective agonists were administrated was not entirely surprising, as abnormal increases in DA transmission should have a higher impact on behaviour when new associations are being established, and be less effective in pre-existing ones^12^,^13^. We did, however, predict both compounds to produce changes in behaviour and alterations in their strategies, as these have been found elsewhere to cause profound changes in the ability to adjust behaviour^51^. The lack of effects on D1a manipulation could also be caused by an abnormal activity of D2 auto-receptors inhibiting DA release^50^. Another possible explanation is that cleaners may already have high endogenous dopamine levels, and thus the addition of an exogenous smaller dosage did not contribute to any further changes in behaviour. However, in human Parkinson’s disease patients, the side effects of significant and long-term increases in dopamine levels are relevant and may include, for instance, excessive risk taking behaviour (pathological gambling)^51^,^61^. Hence, it would be possible for other dosages to be more effective, something that is amenable for further testing in the future.

In this study, we provide first mechanistic evidence regarding the prominence of the dopaminergic system modulation in the context of cooperation (in fish); specifically to the mediation of cleaner wrasses’ negotiation skills and avoidance of negative consequences during potential conflicting interactions. Future studies should also focus on finding out how natural baseline changes in dopamine transmission may influence variation in individual contributions to cooperative behaviour and impulsivity regarding risky choices. While it is important that such studies continue to focus on cleaner wrasses, it is equally relevant to examine other vertebrate model systems, as it has also been demonstrated for humans and other animals^51^,^57^,^61^ particularly in the context of the highly conserved vertebrate social decision-making network^5^,^6^,^7^.

## Materials and Methods

### Field methods

Field experiments took place on reefs around Lizard Island (Lizard Island Research Station, Australia, 14˚40’S, 145˚28’E) with 50 female cleaner wrasses tested between late August and September 2012. Because their larvae settle onto the reef three weeks after hatching^62^ and at Lizard Island this occurs in November and December, their spawning likely occurs between October and December. We thus assumed all sampled females would not have their behaviour affected by this variable. Furthermore, all reefs sampled were fringing reefs, ensuring that all individuals come from ecologically similar contexts. Cleaners were selected haphazardly across reefs, while cleaning stations varied in depth (between 2 and 10 m). All individuals were captured using a barrier net and hand net, measured to the nearest mm (TL – Total Length: ranged from 6.0 cm to 8.1 cm), and their body weight was then estimated using a length-weight regression (Soares MC, unpublished data). Each focal cleaner was administered, via intramuscular injection, with one of five compounds: saline solution for control (0.9% NaCl); a selective D1 agonist SKF-38393 (D047 – Sigma); selective D1 antagonist SCH-23390 (D054 - Sigma); selective D2 (and D3) agonist Quinpirole (Q102 - Sigma); selective D2 antagonist Metoclopramide (M0763 – Sigma). Because this study was done exclusively in field conditions with limitations of time and number of fish used (collecting permit allowance), compound dosages applied were based on previous studies: 5 μg/gbw (gram of estimated body weight) of SKF-38393^63^,^64^,^65^, 0.5 μg/gbw of SCH-23390^52^,^66^, 2μg/gbw of Quinpirole^67^, 5μg/gbw of Metoclopramide^37^,^68^. Injection volumes were always 15 μl per gbw. This process never exceeded 3 min. SKF-38393 is a selective D1 and (and partial D5) agonist that can simulate dopamine activity^63^ and can disrupt collective behaviour, such as shoaling^69^. SCH-23390 is a high-affinity selective D1 antagonist with negligible effects on D2 receptors, and slight effects on 5-HT_2A/C_ receptors^70^. However, effects on the serotonin systems may be dismissed, since 5-HT_2A_ receptors have not been found on fish yet^71^, and the dosage needed to produce effects on 5-HT_2C_ is 10-fold higher than the dosage needed for D1 blockade^72^. Quinpirole is a selective D2 agonist^73^ widely used in a variety of scientific studies related to D2 receptor manipulation. Metoclopramide, commonly known for its anti-emetic effect via the chemoreceptor trigger zone, is a selective D2 antagonist, acting as a dopamine inhibitor^37^,^74^. Although it also has slight effects on the serotonin system, the target receptors have not yet been discovered in the teleost fish brain^71^. Since reward-driven behaviour and decision-making faculties are controlled by central mechanisms, all compounds chosen were reported to be capable of crossing the blood-brain barrier, to ensure the results attained take place in (but not exclusively on) central systems^75^,^76^,^77^,^78^. The order of the treatments was randomized for each dive and all treatments used different cleaner wrasse. After administering an individual it was released and then observed and videotaped for the next 45 min using a Sony Cyber-Shot DSC-W570 camera in a waterproof housing, always from a distance of 2–3 m.

### Behavioural analysis

The following measurements were noted for each interaction filmed on video: a) client species visiting the cleaning station; b) who initiated the interaction: clients were scored as the ones initiating an interaction if they posed before the cleaner touched them. Otherwise, the cleaner was scored as the one initiating; c) duration (in seconds) of inspection towards each client; d) the number and duration of tactile stimulation events provided to each client; and e) number of jolts performed by clients.

### Statistical Analysis

Measures of cleaner wrasse behaviour towards clients were divided into three categories: a) measures of likelihood to engage in cleaning behaviour (motivation to interact); b) measures of interactive investment (provision of tactile stimulation); and c) a measure of cleaner wrasse cheating levels (client jolt rate). The likelihood to engage with clientele was measured by: 1) the proportion of clients inspected (calculated as the total number of clients inspected/total number of visits), and 2) the mean duration of inspection (total time of interaction/total number of interactions). Measures of investment were calculated as: 1) the proportion of interactions in which tactile stimulation was used by cleaners (frequency of clients inspected where tactile stimulation occurred/total number of interactions), and 2) the proportion of time cleaners spent providing tactile stimulation to clients (total tactile stimulation duration/total interaction duration). Finally, the measure of cleaners’ cheating levels was calculated using the frequency of jolts per 100 seconds of inspection. Data were analysed using non-parametric tests because the assumptions for parametric testing were not met. Kruskal-Wallis ANOVA tests were performed to detect differences between treatments for each behavioural variable, and Dunn’s Post-Hoc tests were applied using *Statistica* 12 software, which already include a Bonferroni adjustment to account for multiple comparisons to compare each treatment against the control group^54^. The *p*-value obtained was then further adjusted to account for comparisons against a control group, as suggested by the *Statistica* software^54^.

#### Ethical Note

Ethical clearance to work at Lizard Island Research Station (Australian Museum), which involved animal manipulation, was obtained from The University of Queensland Animal Ethics Committee. The use of animals and data collection complied with the laws of Australia.

#### Data accessibility

The datasets supporting this article have been uploaded as part of the electronic supplementary material.

## Additional Information

**How to cite this article**: Messias, J. P. M. *et al.* Dopamine disruption increases negotiation for cooperative interactions in a fish. *Sci. Rep.* **6**, 20817; doi: 10.1038/srep20817 (2016).
